# Validation and Cultural Adaptation of the Polish Version of the 24-Item Early Onset Scoliosis Questionnaire (EOSQ-24)

**DOI:** 10.3390/jcm14248690

**Published:** 2025-12-08

**Authors:** Edyta Kinel, Krzysztof Korbel, Piotr Janusz, Mateusz Kozinoga, Katarzyna Politarczyk, Karolina Jezierska, Tomasz Kotwicki

**Affiliations:** 1 Department of Rehabilitation, Poznan University of Medical Sciences, 61-545 Poznan, Poland; 2 Department of Physiotherapy, Poznan University of Medical Sciences, 61-545 Poznan, Poland; 3 Department of Spine Disorders and Pediatric Orthopedics, Poznan University of Medical Sciences, 61-545 Poznan, Polandkotwicki@ump.edu.pl (T.K.)

**Keywords:** scoliosis, surveys and questionnaires, quality of life, spine

## Abstract

**Background**: The negative impact of spinal deformities on health-related quality of life (HRQoL) is well known. One goal of early onset scoliosis (EOS) treatment is to improve HRQoL for patients and reduce the burden on the family. The aim of the study was to carry on the process of cultural adaptation of the English version of the Early Onset Scoliosis Questionnaire 24 (EOSQ-24) into Polish. **Methods**: The Polish version of the EOSQ-24 -PL was applied to fifty EOS patients: age 7.5 ± 2.3 years and Cobb angle 24.6 ± 8.5 degrees. The sample included 36 patients with idiopathic, thirteen with congenital, and one with neuromuscular scoliosis. The parental assessment considered each item of the EOSQ-24 relevant to their child’s health condition. EOSQ consists of 24 question items, divided into domains. Transformed scores vary from 0 to 100; lower scores denote a lower quality of life. The reliability (internal consistency, test–retest reliability), floor and ceiling effects, and discriminative validity of the Polish version of the EOSQ-24 were calculated. Internal consistency was assessed using the Cronbach alpha coefficient. Test–retest reliability was evaluated using the intraclass correlation coefficient (ICC_2.1_). **Results**: All items and domains showed very good global internal consistencies (Cronbach’s alpha 0.901 and 0.823, respectively). There was no floor effect, and a ceiling effect ranged from 0 to 62%. The ICC_2.1_ values ranged from 0.74 to 0.98, indicating good to excellent agreement. **Conclusions**: The EOSQ-24-PL is a reliable tool for the psychometric assessment of children with EOS and the family burden.

## 1. Introduction

Early Onset Scoliosis (EOS) is defined as a “scoliosis with onset at less than the age of ten years, regardless of etiology”. The definition of EOS is based upon the consensus from the two study groups: the Growing Spine Study Group (GSSG), the Children’s Spine Study Group (CSSG), and the Scoliosis Research Society (SRS) [1,2,3]. They list four EOS categories: idiopathic, neuromuscular, congenital, and syndromic [2].

EOS curvatures present an increased progression risk, cardiorespiratory problems, and increased morbidity and mortality. EOS management and therapy should take a comprehensive approach, taking into account the psychological impact of EOS on afflicted children’s health-related quality of life (HRQoL) and the stress it creates on their caregivers.

Such an approach remains crucial for analyzing the effectiveness of different treatments or interventions [2].

There are generic (e.g., Pediatric Quality of Life Inventory, Pediatric Evaluation of Disability Inventory, EQ-5D-Y, Strength and Difficulties Questionnaire, Pediatric Outcomes Data Collection Instrument) and disease-specific (e.g., Early-Onset Scoliosis Questionnaire, Shriners Pediatric Instrument for Neuromuscular Scoliosis) outcome measures admitted for the child population, differing by mode of administration depending on the age of the child: patient-reported, parent-reported, parent/caregiver-reported, and/or structured interview/assessment [4].

The disease’s impact and the type of treatment applied to children with spinal deformities are considered multidimensional, encompassing subjective perception, changes in physical, psychological, and social well-being, as well as family burden. The progressive character of spinal deformities can affect children’s HRQoL. Measuring HRQoL in the EOS population requires the use of age-appropriate outcome measures, which is complex, considering the heterogeneity and co-existing health conditions in this group of patients. Assessing HRQoL in populations of patients with EOS is essential, considering the recent, innovative surgical approaches, including growth guidance, designed to reduce the treatment burden. The International Scientific Society on Scoliosis Orthopedic and Rehabilitation Treatment (SOSORT) and the physical and Rehabilitation Medicine section of the European Union of Medical Specialists (UEMS) recommend the overall evaluation of children with spinal deformities using HRQoL questionnaires [5,6].

In the EOS population, the use of a proxy (parent or caregiver) is often required.

In a recent systematic review, Barid et al. analyzed the evidence for the measurement properties of HRQoL outcome measures in the EOS population in a detailed way, supporting the use of the EOSQ-24 among the other questionnaires to evaluate HRQoL outcomes in patients with EOS under the age of 10, highlighting the need for more studies that can strengthen this recommendation [4].

The 24-item Early-Onset Scoliosis Questionnaire (EOSQ-24) was developed by Matsumoto et al. in order to provide a high-quality, consistent assessment of the impact of any EOS treatment technique on HRQoL [7].

The EOSQ-24 is a comprehensive and disease-specific outcome measure [8]. Designed initially in English, its application in varied populations involves translation and cross-cultural adaptation to assure semantic and conceptual equivalence across languages and cultures. Many studies have concentrated on translating and validating the EOSQ-24 in different languages.

These efforts aim to provide a standardised evaluation tool for large patient populations, facilitate multicentre studies, and enhance international communication in the field of EOS research and clinical practice. The EOSQ-24 has been successfully translated and validated in: Spanish, Norwegian, Finish, German, Dutch, Brazilian Portuguese, and Swedish [9,10,11,12,13,14,15], as well as in non-Indo-European languages: Turkish, Chinese (traditional and simplified), Persian (Farsi), Arabic, and Korean [11,16,17,18,19,20,21]; however, it has not been transculturally adapted and validated for Polish patients.

The study aimed to carry on the process of cultural adaptation of the American English version of the EOSQ-24 into Polish.

## 2. Materials and Methods

The a priori hypothesis was proposed as follows: the EOSQ-24-PL questionnaire is a reliable and appropriate tool for the psychometric assessment of children with EOS and the family burden.

### 2.1. Study Population

The Polish version of the EOSQ-24-PL was applied to fifty parents of EOS patients (36 with idiopathic, 13 with congenital, and 1 with neuromuscular scoliosis). The sample included 35 girls and 15 boys. The same multidisciplinary team, consisting of specialists in orthopedics, physiotherapists, and orthopedic technicians, treated the EOS patients. All patients were under conservative treatment.

The mean age of the patients at the time of completing the questionnaire was 7.5 ± 2.3 years, the mean EOS patients’ Cobb angle was 24.6 ± 8.5 degree.

The following criteria for inclusion were applied: (1) patients at the age of 0–10 years; (2) idiopathic, congenital, or neuromuscular scoliosis diagnosis; (3) informed consent obtained from a parent/caregiver; (4) parent/caregiver fluent in Polish (to ensure accurate understanding of the questionnaire); (5) child under regular follow-up in a pediatric orthopedic clinic. Exclusion criteria: (1) patients diagnosed with scoliosis at the age of over 10 years, (2) patients with spinal deformities resulting from trauma or tumor, (3) parents, caregivers who did not agree to sign the Informed Consent Form, (4) parents, caregivers who did not understand Polish.

EOSQ-24-PL was collected from parents of children who visited the outpatient clinic (90%) or the hospital paediatric orthopaedic department (10%).

Parents/caregivers were requested to complete the questionnaire twice a week. In the initial attempt to complete the questionnaire, the time required to respond to each question was measured. To minimize any influence from medical personnel, the participants were placed in a separate area and left to complete the questionnaire independently.

All included children and parents were native Polish speakers. Demographic data, including age, sex, type of scoliosis, and treatment status, were collected during visits to the outpatient clinic or the hospital pediatric orthopedic department.

Before inclusion in the study, informed consent was obtained from parents/caregivers. Data from the EOSQ-24-PL were collected during November 2022 and November 2024.

### 2.2. Early-Onset Scoliosis Questionnaire (EOSQ-24)

EOSQ-24 consists of 24 question items divided into domains: (1) Health-Related Quality of Life, (2) Parental Burden, (3) Financial Burden, and (4) Satisfaction. The Health-Related Quality of Life domain is categorised into eight sub-domains: General Health (2 questions), Pain and Discomfort (2 questions), Pulmonary Function (2 questions), Transfer (1 question), Physical Function (3 questions), Daily Leaving (2 questions), Fatigue (2 questions), Emotion (2 questions). The remaining domains comprise Parental Burden (5 questions), Family Burden (1 question), and domain Satisfaction, which consists of the child’s satisfaction and the parent’s satisfaction (2 questions). The answers to individual questions refer to the period of the last 4 weeks. Questions are scored on a scale of 1 to 5. Using an algorithm: algebraic mean of items answered—1:4 × 100, transformed scores are obtained, ranging from 0 to 100, where lower scores mean greater disability [7].

### 2.3. Adaptation Process

Translation and cross-cultural adaptation of the original EOSQ-24 into Polish were performed according to published guidelines by the International Quality of Life Assessment (IQOLA) [22].

The following steps were used: (1) forward translation, (2) back-translation by an expert panel, (3) pre-testing and cognitive interviewing, and (4) development of the final version.

The sample size was determined using prior validation study recommendations [23].

### 2.4. Forward Translation

Two independent translators converted the original English version into Polish. One of the translators, who had a medical background, was involved in the whole process of adaptation. The second translator had no medical background and was unaware of the project.

### 2.5. Expert Panel Back-Translation

The original and two translated versions were compared at that stage. The two translators and the authors identified differences in translations and produced a combined version. Next—the so-called reversed translation—two translators, native speakers of English, translated the Polish version into the original document’s language (English). Neither translator was familiar with the original version. This stage’s objective was to ensure the equivalence of the two versions and identify possible mistranslations. At the following step, a commission group of translators, a psychologist, a specialist in orthopaedics, and a statistician assessed the translation. The so-called pre-final version was drafted as a result of consensus.

### 2.6. Pre-Testing, Cognitive Interviewing, and Development of the Final Version

Fifty EOS patients (who met the study eligibility requirements described above) were checked for the pre-final questionnaire’s comprehensiveness to ensure the adapted version was understandable. The parents were interviewed after completing the questionnaire to discuss their interpretation of each question and answer. Then, the committee reassessed this test’s outcome, and the final form of the questionnaire was created (Supplemental Data File S1).

### 2.7. Statistical Analyses

All statistical analyses were carried out using the Real Statistics Resource Pack software (Release 9.4) with a significance level of α < 0.05.

### 2.8. Reliability

Reliability was assessed using the two most important properties: consistency and stability.

Internal consistency was assessed using Cronbach’s alpha coefficient. Cronbach’s alpha ranges from 0 (none of the items are correlated with one another) to 1 (reflects perfect internal consistency). Cronbach’s alpha of at least 0.70 has been chosen to indicate adequate internal consistency [23].

Test–retest reliability was evaluated using the intraclass correlation coefficient (ICC_2.1_, CI = 95%), the most suitable and commonly used reliability parameter for ordinal measures. ICC_2.1_ concerns the variation in the population (interindividual variation) divided by total variation, which is the interindividual variation plus the intraindividual variation (measurement error), expressed as a ratio between 0 and 1. The sum of scores obtained for each question provided by all parents during the first and second times, and the sum of total scores obtained for each parent, were used, respectively, for test–retest analysis. A positive rating for reliability is when the ICC_2.1_ is at least 0.70 in a sample size of at least 50 patients [23,24].

A second survey was sent by e-mail 7 days after the initial assessment.

### 2.9. Measurement Error

The standard error of measurement (SEM) and minimal detectable change at the 90% level (MDC90) were employed. The sample included all parents of EOS patients (*n* = 50) who completed the EOSQ-24-PL twice. The SEM was calculated as an element from the mean square error (MSE) from the analysis of variance, ANOVA. The MDC is the minimum change in a patient’s score that ensures the change is not the result of measurement error. The MDC90 was calculated using the formula: MDC = SEM × 1.65 × √2, where 1.65 is the z-value that reflects the 90% CI of no change [25] and √2 indicates two measurements assessing change [25].

### 2.10. Floor and Ceiling Effects

Floor and ceiling effects were calculated and considered to be high if >15% of parents of EOS patients reported the worst or the best status, respectively [24].

The distribution of results indicates the number (percentage) of patients with a minimum score (floor effect) and the number (percentage) of patients with a maximum score (ceiling effect).

### 2.11. Discriminative Validity

Discriminative validity for scoliosis etiology was tested using the Mann–Whitney test.

## 3. Results

The Polish version of the EOSQ-24-PL was applied to fifty parents of patients with EOS: the patients’ age range was from 2 to 10, with a mean of 7.5 ± 2.3 years, and the mean Cobb angle was 24.6 ± 8.5 degrees. The sample included thirty-six patients with idiopathic scoliosis, thirteen with congenital scoliosis, and one with neuromuscular scoliosis (Table 1).

The assessment of parents considered each item of the EOSQ-24-PL relevant to their child’s health condition. The duration of completing the questionnaire was ≤10 min for all parents. All questionnaires were returned without any missing data.

The mean score of the EOSQ-24-PL was 75.81 ± 11.39. The lowest scoring domains were Financial Burden (40.0 ± 18.21) and Parental Burden (69.4 ± 18.03). Considering subdomains, the subdomain General Health (63.3 ± 15.85) resulted in the lowest score (Table 2).

### 3.1. Reliability

All items (Table 3) and domains showed excellent or good global internal consistencies (Cronbach’s alpha 0.901 and 0.823, respectively).

In domain analysis, the lowest Cronbach alpha was for the Parental Burden domain (0.786) and the highest for the Health-Related Quality of Life (0.84) and Financial Burden (0.834) domains.

Test–retest reliability for 24 items ranged from 0.74 to 0.98 ICC values, indicating good to excellent agreement. In domain analysis, ICC values were from 0.90 for the Financial Burden domain to 0.94 for the Satisfaction and 0.93 Health Related Quality of Life domains.

### 3.2. Floor and Ceiling Effects

No floor effect >15% was seen. The floor effect was from 0 to 2% in the Financial Burden domain. The ceiling effect ranged from 0 to 10% in the domains’ data: Health-Related Quality of Life and Financial Burden (0%), Parental Burden (4%), and Satisfaction (10%). In subdomains, the data ceiling effect was as follows: General Health (2%), Pain and Discomfort (26%), Pulmonary Function (56%), Transfer (62%), Physical Function (52%), Daily Leaving (42%), Fatigue (10%), Emotion (20%).

### 3.3. Discriminative Validity

The mean score of the EOSQ-24-PL was 76.67 ± 11.27 in the idiopathic group and 73.63 ± 12.29 in the congenital group.

There was a statistically significant difference in answers between the idiopathic and congenital groups in the Daily Living and Emotion subdomains.

In correlation with the major curve and domains, there was no significant difference in the idiopathic and congenital groups.

Considering subdomains, the major curve was significantly correlated with Physical Function, Daily Living, and Fatigue subdomains in the congenital group. There was no significant difference in the idiopathic group.

## 4. Discussion

This study presents a Polish adaptation of the EOSQ-24-PL, a new measure of HRQoL in EOS patients.

### 4.1. Statistical Relevance

Similarly to previous cross-cultural adaptations, the lowest mean values of EOSQ-24 were observed in the following domains: Financial Burden [18], Parental Burden domains [17,18], and General Health subdomain [9,18].

The EOSQ-24-PL had an excellent value of Cronbach’s alpha coefficient (0.901), exceeding the minimum recommended value of 0.70 and indicating satisfactory internal consistency as a factor of satisfactory reliability of the EOSQ-24-PL. Our results revealed good or acceptable internal consistency for the domains. The Cronbach’s alpha score was as high as that achieved by the authors of the original version, 0.92 [7].

These results are similar to previous cross-cultural adaptations with an excellent [10,11,12,13,15,16,18,19,21] or good value of Cronbach’s alpha coefficient [9,14,17,20].

Test–retest reliability indicated good to excellent agreement, as in previous studies [10,11,13].

There was no floor effect, while there were marked ceiling effects in the following subdomains: Pain and Discomfort (26%), Pulmonary Function (56%), Transfer (62%), Physical Function and Daily Living (52%), which can be the result of a moderate Cobbe value and a conservative approach to treatment. In previous studies, the ceiling effect was more often noted than the floor effect [9,10,11,12,13,15,16,17,18,20]. The described effects (floor or ceiling) may be a potential source of error during statistical analysis when comparing more treatment groups [26].

### 4.2. Clinical Relevance

The natural history of EOS indicates that earlier onset generally leads to worse final curvature and prognosis. The cutoff of 10 years for EOS is essential because the most rapid growth of alveoli in the lungs occurs during the first 8 years of life [27]. Additionally, a significant growth of the spine and thorax happens before age 10 [28].

Treatment alternatives for EOS aim to correct spinal and thoracic deformity, improve respiratory function, and enhance HRQoL [29].

Ramo et al. underlined “the significance of the etiology of EOS on the parent-reported HRQoL outcomes of the disease. Specifically, syndromic and neuromuscular EOS diagnoses are associated with lower EOSQ-24 scores before treatment compared to congenital and idiopathic diagnoses. Radiographic measurements of severity have a relatively small influence on EOSQ-24 scores. These baseline differences in EOS-designated etiology should be accounted for in studies comparing treatment outcomes for this heterogeneous patients’ population” [8].

These results are also confirmed in other studies in which the total score of the EOS-24 questionnaire decreased with increasing etiology complexity. Patients with neuromuscular or syndromic scoliosis had a significantly lower score than those with idiopathic or congenital scoliosis. EOS patients who were before surgery had a significantly lower total score than children who were conservatively treated [10,11].

In our study, in correlation with the major curve and domains, there was no significant difference in the idiopathic and congenital groups. It can be a result of both a relatively small group of patients and a moderate Cobb angle, as well as the fact that all patients were undergoing conservative treatment. Considering all subdomains of the EOS-24 questionnaire, in our patient group, the major curve was significantly correlated with the Physical Function, Daily Living, and Fatigue subdomains in the congenital group. There was no significant difference in the idiopathic group. These results confirm the influence of the etiology of EOS and the length of treatment on the health status of the patients.

In our study, the sample size mean age was 7.5 ± 2.3 years, the patients were under conservative treatment, while four were before planned surgery. Similar groups of EOS patients below 10 years old were analysed in other studies [10,11,13,16,20,21].

The EOSQ-24 was completed by 90% of mothers of patients and only by 10% of fathers. As it was noticed in the study by Hwang et al., considering that caregiving roles may be influenced by sex, it would be interesting to analyse in the future the possibility of different responses to specific items of the questionnaire answered by males versus females [21].

Moreover, during the analysis of the EOSQ-24 answers given by the caregivers, it is recommended that they evaluate it from their perspective, which can be different from the functional assessment made by a medical practitioner [7]. A study conducted by Gotlieb et al., analysing proxy versus patient perception (older children) questionnaires, also confirms that, for developmentally neurotypical older children and adolescents, self-reported outcomes are recommended. When the EOSQ-24 (caregiver-reported) was compared synchronously with the SRS-22 questionnaire (patient self-reported, used in older neurotypical children), a strong correlation was found only for the Pain domain. All the above suggest that caregivers and older children may not share the same perspective on other domains of HRQoL [30].

EOS develops significant physical and physiological impacts, and the psychological burden on patients must also be considered, as it affects their HRQoL [31]. The natural progression of physical deformity reveals aesthetic consequences that impact self-esteem. Patients often need lifestyle adjustments. In patients with EOS, depression and anxiety are more prevalent [32].

Self-reported questionnaires should be used when possible to assess HRQoL in older children and adolescents with EOS who are developmentally neurotypical [30]. A multidisciplinary approach that considers this group of patients’ psychological impacts and needs seems essential [29], considering the individual interpretation of the answers given by individual respondents.

The study’s clinical relevance lies in the need to incorporate changes in HRQoL during the evaluation and monitoring of overall treatment results in patients with EOS. Using a reliable HRQoL tool by all specialists involved in the treatment process will simplify the collection of individual results and their objective interpretation. Moreover, it provides the following general recommendations for treating children with EOS, one of the primary aims of which is to enhance the quality of life for this sensitive group.

The evolving healthcare environment and societal trends have a significant impact on the management of early-onset scoliosis. The increasing availability of highly specialized professionals and dedicated rehabilitation centers enables more personalized and multidisciplinary care, which aligns with the holistic approach recommended by international guidelines, such as SOSORT. At the same time, changing lifestyles and widespread access to social media shape patient and caregiver expectations. Families are now more informed about treatment options and often compare outcomes through online support groups, which can provide psychological support but also create stress related to aesthetic concerns and perceived treatment success. These dynamics underscore the importance of incorporating HRQoL assessment into routine practice—not only to monitor physical outcomes but also to address emotional well-being and family burden in an era where quality of life is as critical as radiographic effects.

Our findings support the use of the EOSQ-24 to evaluate HRQoL outcomes in patients with EOS. At the same time, confidence in this recommendation is mitigated by low-quality evidence. It should be strengthened by further studies of the EOSQ-24 measurement properties, considering the heterogeneity and spectrum of severity within EOS patients [4].

### 4.3. Study Limitations

The first limitation of our study is a relatively lower sample size compared to other analysed samples of EOS patients. Secondly, in the sample, there was only one EOS patient with neuromuscular scoliosis, so it was impossible to analyse the differences among more etiologies of deformities. Thirdly, patients were only conservatively treated. Investigating these specific aspects with a larger sample size is under our ongoing study. Finally, in the future, the Polish version of the EOSQ-24-PL questionnaire could be tested on parents of patients who had undergone surgical treatment.

## 5. Conclusions

The Polish version of the EOSQ-24-PL is a reliable and valid tool for the psychometric assessment of children with EOS and the family burden.

### Clinical Significance

The EOSQ-24-PL can be applied in routine clinical practice to monitor the effectiveness of treatment, in this particularly demanding group of patients with Early Onset Scoliosis. Additional information about the HRQoL of individual patients, obtained from the questionnaire, enabled the multidisciplinary team involved in the treatment process to gain a better understanding of how the type of treatment affects the health status of the EOS patient and the family burden from the parents’ or caregivers’ perspective. HRQoL assessment provides more than statistical validation; it influences therapeutic strategies and family well-being. Identifying domains with low scores may prompt clinicians to adjust treatment plans, introduce psychological support, or consider earlier surgical intervention. Moreover, EOSQ-24 captures parental and financial burden, which is essential for planning multidisciplinary care and offering social or psychological assistance. Incorporating HRQoL monitoring into routine practice ensures that treatment goals extend beyond clinical and radiographic correction to improve overall quality of life for both patients and their families.

Moreover, using the same HRQoL tool, validated in multiple languages, could constitute the basis for creating a multicenter study to better understand the changes in HRQoL of the EOS population during the treatment of a larger population with diverse cultural backgrounds and environments.

## Figures and Tables

**Table 1:** Patients Characteristics.

Variable Value	Value
Number of patients (*n*)	50
Age (yr)	7.5 ± * 2.3
**Sex**	
Female	35
Male	15
**Diagnosis**	
Idiopathic	36
Congenital	13
Neuromuscular	1
Cobb angle (°)	24.6 ± * 8.5
**Treatment state**	
Physiotherapy	17
Physiotherapy and Brace	25
Brace	4
Before surgery	4
Mean time of treatment (yr)	2.32 ± * 1.8

* standard deviation SD (±), degree (°).

**Table 2:** The scores obtained in domains/subdomains using the EOSQ-24-PL.

Domains/Subdomains EOSQ-24-PL	Mean * (±) SD
Health-Related Quality Of Life	80.6 (±) 10.82
- General Health	63.3 (±) 15.85
- Pain and Discomfort	76.3 (±) 18.43
- Pulmonary Function	91.8 (±) 12.78
- Transfer	91.0 (±) 15.78
- Physical Function	91.0 (±) 14.85
- Daily Living	83.0 (±) 23.66
- Fatigue	73.5 (±) 17.25
- Emotion	75.3 (±) 17.94
Parental burden	69.4 (±) 18.03
Financial burden	40.0 (±) 18.21
Satisfaction	71.3 (±) 18.77

* standard deviation SD (±).

**Table 3:** The Cronbach’s alpha value coefficient of the EOSQ-24-PL compared to the EOSQ-24 (original).

EOSQ-24-PL	EOSQ 24 (Original)
Cronbach’s alpha	Cronbach’s alpha
0.901	0.92

## Data Availability

The data presented in this study are available upon request from the corresponding author.

## Supplementary Materials

The following supporting information can be downloaded at: https://www.mdpi.com/article/10.3390/jcm14248690/s1, Supplemental Data File S1: EOSQ-24-PL questionnaire.

## Abbreviations

The following abbreviations are used in this manuscript:

EOS	Early Onset Scoliosis
HRQoL	Health-Related Quality of Life
EOSQ 24	Early Onset Scoliosis Questionnaire 24
EOSQ-24-PL	Early Onset Scoliosis Questionnaire 24 Polish version
ICC	Intraclass Correlation Coefficient
GSSG	Growing Spine Study Group
CSSG	Children’s Spine Study Group
SRS	Scoliosis Research Society
SEM	Standard Error of Measurement
MDC90	Minimal Detectable Change at the 90%
MSE	Mean Squared Error
