# Supplementary material for: Validation and Cultural Adaptation of the Polish Version of the 24-Item Early Onset Scoliosis Questionnaire (EOSQ-24)
Source: J Clin Med. 2025 Dec 8;14(24):8690. doi: 10.3390/jcm14248690 (PMC12734251; doi:10.3390/jcm14248690)
Supplement: Supplementary file 1 [file jcm-14-08690-s001.zip › Supplemental Data File S1.pdf]

Kwestionariusz jakości życia dla skoliozy dziecięcej (EOSQ 24-PL)

Department of Spine Disorders and Pediatric Orthopedics, University of Medical Sciences in Poznan, Poland
Department of Physiotherapy, University of Medical Sciences in Poznan, Poland
Department of Rehabilitation, University of Medical Sciences in Poznan, Poland

Ogólny stan zdrowia: <u>W ciągu ostatnich 4 tygodni</u>				
**1. Jak, ogólnie ujmując, ocenia Pani/Pan stan zdrowia swojego dziecka:**				
Słaby	Dość dobry	Dobry	Bardzo dobry	Doskonały
**2. Jak często choruje Pani/Pana dziecko?**				
Stale	Przez większość czasu	Od czasu do czasu	Rzadko	Wcale

Ból/dyskomfort : <u>W ciągu ostatnich 4 tygodni</u>				
**3. Jak często dziecko skarży się na ból/dyskomfort?**				
Stale	Przez większość czasu	Od czasu do czasu	Rzadko	Wcale
**4. Jak silny jest ból/dyskomfort dziecka?**				
Bardzo silny	Silny	Umiarkowany	Łagodny	Nie występował

Czynność płuc: <u>W ciągu ostatnich 4 tygodni</u>				
**5. Czy trudno dziecku jest mówić/gaworzyć/płakać (stosownie do wieku) bez odczuwania duszności?**				
Trudno	Raczej trudno	Ani łatwo, ani trudno	Raczej łatwo	Łatwo
**6. Jak często dziecko odczuwa duszności podczas codziennej aktywności?**				
Stale	Przez większość czasu	Od czasu do czasu	Rzadko	Wcale

Przemieszczanie się: <u>W ciągu ostatnich 4 tygodni</u>				
**7. Jak często stan zdrowia dziecka ogranicza mu dostępność do różnych miejsc?**				
Stale	Przez większość czasu	Od czasu do czasu	Rzadko	Wcale

Funkcjonowanie: <u>W ciągu ostatnich 4 tygodni</u>				
**8. Czy trudno jest dziecku poruszać górną częścią ciała (głowa, klatka piersiowa, barki, ręce)?**				
Trudno	Raczej trudno	Ani łatwo, ani trudno	Raczej łatwo	Łatwo
**9. Czy samodzielne siedzenie jest dla dziecka trudne?**				
Trudne	Raczej trudne	Ani łatwe, ani trudne	Raczej łatwe	Łatwe
**10. Czy utrzymanie równowagi podczas raczkowania, chodzenia lub biegania jest dla dziecka trudne?**				
Trudne	Raczej trudne	Ani łatwe, ani trudne	Raczej łatwe	Łatwe

CIĄG DALSZY NA STRONIE NASTĘPNEJ

**Życie codzienne: <u>W ciągu ostatnich 4 tygodni</u>**				
**11. Czy samodzielne ubieranie się dziecka jest dla niego trudne lub czy wymaga pomocy w ubieraniu? (np. zdejmowanie/zakładanie ubrań, wkładanie rąk i nóg w rękawy i nogawki, pomoc przy guzikach, zapinkach, zamkach, zatrzaskach, rzepach)**				
Trudne	Raczej trudne	Ani łatwe, ani trudne	Raczej łatwe	Łatwe
**12. Moje dziecko potrzebuje więcej czasu niż zdrowe dzieci na zjedzenie takiej samej porcji.**				
Zdecydowanie tak	Raczej tak	Ani tak, ani nie	Raczej nie	Zdecydowanie nie

**Poziom zmęczenia/energii: <u>W ciągu ostatnich 4 tygodni</u>**				
**13. Jak często dziecko odczuwa zmęczenie?**				
Stale	Przez większość czasu	Od czasu do czasu	Rzadko	Wcale
**14. Jak trudno jest być dziecku aktywnym przez cały dzień?**				
Trudno	Dość trudno	Ani łatwo, ani trudno	Dość łatwo	Łatwo

**Emocje: <u>W ciągu ostatnich 4 tygodni</u>**				
**15. Jak często dziecko odczuwa niepokój/rozdrażnienie z powodu swojego stanu zdrowia?**				
Stale	Przez większość czasu	Od czasu do czasu	Rzadko	Wcale
**16. Jak często Twoje dziecko odczuwa frustrację z powodu swojego stanu zdrowia?**				
Stale	Przez większość czasu	Od czasu do czasu	Rzadko	Wcale

**Wpływ na rodziców: <u>W ciągu ostatnich 4 tygodni</u>**				
**17. Czy często odczuwa Pani/Pan obawy/niepokój o stan zdrowia dziecka?**				
Stale	Przez większość czasu	Od czasu do czasu	Rzadko	Wcale
**18. Jak często stan zdrowia dziecka zaburza aktywności rodzinne?**				
Stale	Przez większość czasu	Od czasu do czasu	Rzadko	Wcale
**19. W jakim stopniu czuje się Pani/Pan wyczerpana/y z powodu stanu zdrowia dziecka?**				
Ekstremalnie	Bardzo	Umiarkowanie	Nieznacznie	Wcale
**20. Jak często zdarza się Pani/Panu opuszczać lub spóźniać do pracy lub na spotkania towarzyskie z powodu stanu zdrowia dziecka?**				
Stale	Przez większość czasu	Od czasu do czasu	Rzadko	Wcale
**21. Czy pomimo stanu zdrowia dziecka jest Pani/Pan w stanie spędzać wystarczająco dużo czasu z rodziną/partnerem/małżonkiem/małżonką?**				
W ogóle	Niewiele	Od czasu do czasu	Przez większość czasu	Stale

CIĄG DALSZY NA STRONIE NASTĘPNEJ

Konsekwencje finansowe: <u>W ciągu ostatnich 4 tygodni</u>

22. Jak dużym obciążeniem finansowym dla Pani/Pana jest skolioza u dziecka?

Ekstremalnym	Dużym	Umiarkowanym	Niewielkim	Nie jest Obciążeniem

Poziom satysfakcji: <u>W ciągu ostatnich 4 tygodni</u>

23. Czy dziecko jest zadowolone ze swojej sprawności?

Bardzo niezadowolone	Niezadowolone	Ani tak, ani nie	Zadowolone	Bardzo zadowolone

24. Czy <u>Pani/Pan</u> jest zadowolona/y ze sprawności dziecka?

Bardzo niezadowolona(y)	Niezadowolona(y)	Ani tak, ani nie	Zadowolona(y)	Bardzo zadowolona(y)

DZIĘKUJEMY
